# Iron Porphyrin-Based Composites for Electrocatalytic Oxygen Reduction Reactions

**DOI:** 10.3390/molecules29235655

**Published:** 2024-11-29

**Authors:** Stennard Leetroy George, Linkai Zhao, Ziyi Wang, Zhaoli Xue, Long Zhao

**Affiliations:** School of Chemistry and Chemical Engineering, Jiangsu University, Zhenjiang 212013, China; stennardgeorge@gmail.com (S.L.G.); 2212312055@stmail.ujs.edu.cn (L.Z.); 2212312054@stmail.ujs.edu.cn (Z.W.)

**Keywords:** oxygen reduction reaction, electrocatalysts, iron porphyrins, axial ligands, substituent effects, functional group effects

## Abstract

The oxygen reduction reaction (ORR) is one of the most critical reactions in energy conversion systems, and it facilitates the efficient conversion of chemical energy into electrical energy, which is necessary for modern technology. Developing efficient and cost-effective catalysts for ORRs is crucial for advancing and effectively applying renewable energy technologies such as fuel cells, metal–air batteries, and electrochemical sensors. In recent years, iron porphyrin-based composites have emerged as ideal catalysts for facilitating effective ORRs due to their unique structural characteristics, abundance, advances in synthesis, and excellent catalytic properties, which mimic natural enzymatic systems. However, many articles have focused on reviewing porphyrin-based frameworks or metalloporphyrins in general, necessitating research specifically addressing iron porphyrin. This review discusses iron porphyrin as an effective catalyst in ORRs. It provides a comprehensive knowledge of the application of iron porphyrin-based composites for electrocatalytic ORRs, focusing on their properties, synthesis, structural integration with conductive supports, catalytic mechanism, and efficacy. This review also discusses the challenges of applying iron porphyrin-based composites and provides recommendations to address these challenges.

## 1. Introduction

The majority of the energy that is consumed worldwide is produced through the combustion of fossil fuels [1]. The extensive use of fossil fuels for industrial and economic development has birthed an energy crisis and many environmental issues, such as global warming, climate change, and air pollution. For instance, when fossil fuels are combusted, harmful by-products (pollutants) such as nitrogen oxide (NO_x_), sulfur dioxide (SO_x_), volatile organic compounds (VOCs), and particulates are produced, which all have deleterious effects on the environment [2,3]. Hence, using fossil fuels negatively affects the environment and the world’s economy and threatens human health [4,5,6]. To mitigate the adverse environmental effects and produce clean, sustainable energy, advancing renewable energy technology that produces low carbon emissions is essential. Among advanced technologies, polymer electrolyte membrane fuel cells (PEMFCs) and metal–air batteries are promising for powering automotive and household appliances [7,8,9,10]. Compared to internal combustion engines, PEMFCs can produce electrical energy with an impressive efficiency of 40–60% [11,12]. Metal–air batteries, for their part, generate electricity through chemical reactions between a metal (such as zinc) and oxygen from the air, where the metal acts as the anode and the oxygen acts as the cathode for the chemical reaction. Metal–air batteries offer an eco-friendly option and achieve energy efficiency levels that have a five to thirty times higher energy efficiency than lithium-ion batteries [13]. They are perceived as a sustainable energy alternative due to their eco-friendly, non-toxic, and low-cost nature, and they are a viable alternative as metals are abundant in nature [14,15]. These technologies utilize ORRs to convert chemical energy into electrical energy [16,17]. For example, a typical H_2_/O_2_ PEMFC oxidizes hydrogen (H_2_) at the anode, producing protons (H^+^) and electrons (e^−^). This is known as the hydrogen oxidation reaction (HOR), as seen below [18,19,20].

H_2_ → 2e^−^ + 2H^+^ (1)

Electrons produced from HOR reactions are moved from the anode to the cathode to participate in the ORR, while the protons travel through the proton exchange membrane (PEM) and flow into the cathode’s electrolyte. Oxygen combines with the protons and electrons at the cathode to produce water.

The ORR process occurs at the cathode and involves multiple steps, generally by two major pathways: a four-electron (4e^−^) pathway from O_2_ to H_2_O or a two-electron (2e^−^) pathway from O_2_ to H_2_O_2_/HO_2_^−^, depending on the nature of electrolyte. The following equations show that these reactions occur in acidic and alkaline media [21,22,23,24,25].

In an acidic medium, the equation is as follows:

O_2_ + 4H^+^ + 4e^−^ → 2H_2_O (2)

O_2_ + 2H^+^ + 2e^−^ → H_2_O_2_ (3)

H_2_O_2_ + 2H^+^ + 2e^−^ → 2H_2_O (4)

In an alkaline medium, the equation is as follows:

O_2_ + 2H_2_O + 4e^−^ → 4OH^−^ (5)

O_2_ + H_2_O + 2e^−^ → HO_2_^−^ + OH^−^ (6)

HO_2_^−^ + H_2_O + 2e^−^ → 3OH^−^ (7)

These equations show that in an acidic environment, O_2_ can be converted to the H_2_O_2_ intermediate via the 2e^−^ pathway or directly to H_2_O through the 4e^−^ pathway. In an alkaline environment, the products are HO_2_^−^ or H_2_O, depending on the electron pathway. The reason for the difference between the intermediate produced in the acid and alkaline electrolytes is the source of protons. Protons in acidic media come directly from the hydronium ion (H_3_O^+^), while protons originate from the decomposition of water in alkaline media [26,27].

These reactions are fundamental to the operation of the fuel cell, where the four-electron (4e^−^) transfer pathway attains maximum energy conversion efficiency [25]. The 2e^−^ ORR pathway cannot liberate maximum energy because the process generates intermediates capable of blocking the active sites at the catalyst surface and destroying the catalyst surface due to oxidation of the carbon framework, thus drastically reducing the ORR performance.

These ORRs are kinetically sluggish and require an electrocatalyst to lower the activation energy needed to break the stable O=O bond (D_O=O_ = 498 kJ mol^−1^) [28]. Therefore, there is a need for a highly efficient and selective catalyst to enhance the kinetics of the electrochemical process. Studies have shown that platinum-based materials are efficient for ORRs. Pt has excellent catalytic activity and is selective towards the thermodynamically favorable 4e^−^ pathway. It has a proven performance, where practical applications have demonstrated its reliability and consistency in catalyzing fuel cell reactions [29,30,31]. This makes platinum catalysts the benchmark for ORR catalysts, but they are costly, have limited availability, and are short-lived [19,29,30]. Moreover, platinum-based electrocatalysts are usually susceptible to fuel crossover when using alcohols, thus decreasing the fuel cell’s efficiency and performance [32,33]. Fuel crossover refers to the unintended migration of reactants, such as methanol, across the electrolyte membrane from the anode to the cathode. When these reactants cross, they can react directly with oxygen at the cathode, reducing potentials because the catalysts will have to deal with the unintended oxidation of the crossover fuel. Furthermore, the crossover fuel can cause poisoning and degradation of the catalyst, reducing the longevity of the fuel cell [28,34,35].

Additionally, platinum catalysts are scarce and are associated with price pressures due to increasing demand [36]. Numerous green technologies that depend on Pt electrocatalysts have found it difficult to become commercially viable because of these issues [37]. The challenge is to develop alternative catalysts that offer the same high efficiency and selectivity at a lower cost and with more excellent stability [38,39].

**Figure 1:**
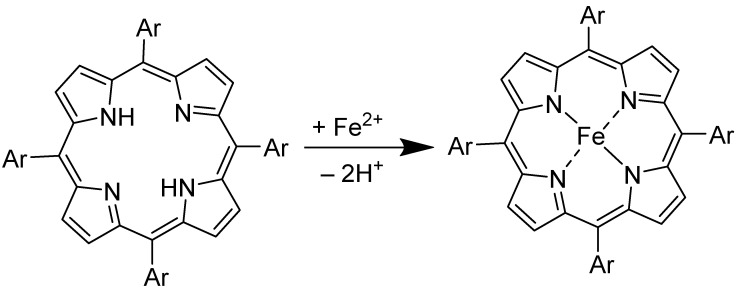
Iron coordination in porphyrin.

**Figure 2:**
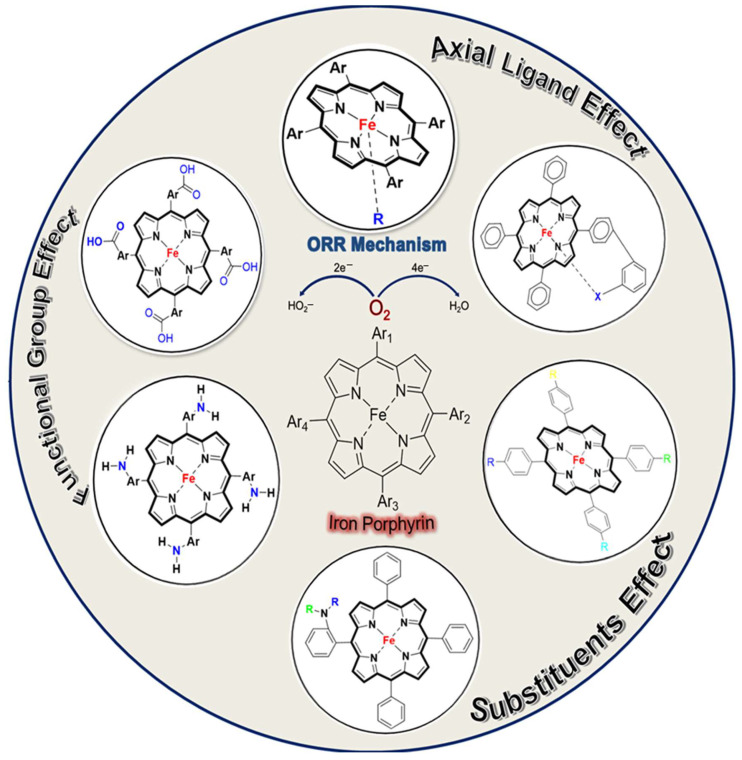
The scope of the review.

Iron porphyrins appear to be an excellent alternative to platinum. They have garnered much attention because of their catalytic efficiency, structural versatility, and mechanistic innovation [40,41]. A typical iron porphyrin is a metalloporphyrin complex in which an iron ion is coordinated and held within a porphyrin ring, as shown in Figure 1 [42]. This porphyrin ring is a large, aromatic macrocycle composed of four pyrrole subunits connected via methine bridges (=CH-) [43]. In iron porphyrins, the iron ion binds to the pyrrole central nitrogen atoms of the inner porphyrin ring, resulting in a robust, coordinated iron–nitrogen (Fe-N_4_) chemical environment [27,44]. The central metal can exist in various oxidation states, most commonly Fe^2+^ or Fe^3+^, and it can bind other ligands, thus making it an essential component in different biological and synthetic systems. Iron porphyrin complexes, such as heme, are present in proteins and enzymes. These serve in oxygen binding and transport in hemoglobin, oxygen storage in myoglobin, and electron transfer in cytochromes b and c [45,46]. Also, it is necessary for oxygen activation in cytochromes oxidase and the catalytic breakdown of peroxide in peroxidase and catalase [47,48,49]. The varied functions of heme in O_2_ and H_2_O_2_-related processes are underpinned by their finely modifiable functions based on the chemical environment and the type of ligands present [38,42,47]. For instance, cytochrome c oxidase activates oxygen at its heme iron porphyrin site, initiating a process of breaking the O=O bond. This action creates a crucial intermediate, the Fe (IV)-oxo porphyrin cation radical, which is essential in reducing oxygen to water via the 4e^−^ route [50,51,52]. As electrocatalysts, iron porphyrin complexes can facilitate ORRs at lower overpotentials than other catalysts. The ORR efficiency through the 4e^−^ path can be optimized by altering the electronic properties of the iron porphyrin, which is achieved through the precise tuning of the porphyrin ring. Introducing various axial ligands, functional groups, or substituents allows for such modifications, which influence the electronic environment around the iron center. As illustrated in Figure 2, these effects, including axial ligand, functional group, and substituent modifications, are key to enhancing ORR performance. These structural adjustments offer deeper insight into the electron transfer processes and allow for better control over the reaction kinetics and selectivity [53].

Furthermore, investigating the ORR mechanism involving iron porphyrins helps in understanding the catalytic process’s fundamental aspects. This knowledge can guide the design of more effective catalysts by revealing the steps involved in the oxygen reduction pathway and identifying possible intermediates. Moreover, iron is an abundant and relatively inexpensive metal, making iron porphyrin-based catalysts economically attractive for large-scale applications relative to precious metals like platinum. Thus, developing iron porphyrin-based electrocatalysts is vital for advancing sustainable energy technologies.

This review examines the current state of research on iron porphyrin-based composites as electrocatalysts in applications for ORRs. It also evaluates the efficacy and mechanisms of the iron porphyrin-based composites for ORRs. Although many articles have reviewed porphyrins-based frameworks or metalloporphyrins in general, this article will look specifically at research concerning iron porphyrin complexes. 

For example, Cheng, N. et al. (2012) provide an overview of the advancements in using metalloporphyrin catalysts for ORR in fuel cells and cover modifications such as metal oxide co-depositions and the use of various supports (e.g., carbon and gold) [54]. The group’s paper focuses heavily on the fundamental aspects of porphyrin-based catalysts for ORR in fuel cells and the need for cheaper alternatives to platinum. However, it leaves the discussion on practical, large-scale applications relatively undeveloped. This review article expands on this idea by focusing specifically on iron porphyrins, which are cheaper and more abundant, and emphasizes the practical aspects of scalability and stability in real-world conditions. These are critical for the commercial viability of ORR catalysts in fuel cells and metal–air batteries. Likewise, Hua, Q. et al. (2022) discuss the effects of different support materials on the ORR activity of an iron porphyrin-based catalyst [55]. Their paper covers a range of support materials rather than concentrating on the iron porphyrin catalyst. This article captures the specific nuances of iron porphyrins, including how structural adjustments influence performance, which is crucial for advancing ORR catalysis. Chatterjee, S. et al. (2017) investigate the mechanistic details of iron porphyrin catalysis and emphasize the role of axial ligands and second-sphere interactions in influencing the rate of O=O bond cleavage and overall reaction pathways [56]. This article further summarizes these mechanisms and focuses on the practical implications and challenges of scaling iron porphyrins for real-world applications. Furthermore, the ORR mechanism is examined in both acidic and alkaline media, which is valuable for understanding their performance across different operating environments. Xie, L. et al. present a synthetic iron porphyrin complex inspired by natural enzymes designed to enhance both ORR and oxygen evolution reactions (OERs) [57]. Like Xie, L., this article addresses the use of axial ligands but takes a more targeted approach, exploring other modifications such as meso-substituents, functional groups, and heteroatom doping. These are key to improving the ORR performance in practical applications. 

By narrowing the scope to iron porphyrin-based catalysts, the review fills the gap left by studies that either generalize catalyst types or overlook iron porphyrins in detail. Herein, the electrocatalytic mechanisms in acid and alkaline electrolytes for ORRs are discussed using iron porphyrin models. The best structures of iron porphyrin composites and their performances under various conditions are examined. The challenges and solutions are highlighted for these electrocatalysts’ future development and practical application.

## 2. Electrocatalytic Mechanism for ORRs

### 2.1. Evaluation of the ORR Mechanism

The electrocatalytic mechanism for ORRs is comprehensively studied by employing the linear sweep voltammetry (LSV) technique, including both the rotating ring disk electrode (RRDE) and the rotating disk electrode (RDE) measurements. Both methods are conducted using the standard three-electrode setup, which includes a saturated silver/silver chloride (Ag/AgCl) electrode as the reference electrode, a platinum (Pt) wire/mesh/net as the counter electrode, and a catalyst-coated glassy carbon (GC) electrode as the working electrode. Measurements are usually performed in acidic and alkaline electrolytes purged with O_2_ or N_2_. The O_2_- and N_2_-saturated environments are mainly used to reduce the impact of the double-layer capacitance during measurements [58].

The LSV measurements in O_2_-saturated electrolytes evaluate key performance parameters critical for understanding the reaction mechanism and evaluating ORR efficiency. The first parameter is current density, which relates to the kinetics of the reaction and assesses overall activity. Tafel equations are used to convey the electrochemical reaction rate to the overpotentials. The second and third parameters are the onset potential (E_onset_) and the half-wave potential (E_½_), respectively, which are obtained from the LSV curve at a specific rotation [38,52]. The onset potential has several definitions, varying between published works. One definition determines the E_onset_ at the inflection point on the LSV curve [38]. The inflection point on a voltammogram signifies a transition in the reaction system, corresponding to the change in the curvature of the graph. This definition usually refers to the point of the most intense increase in the current rate, which implies a quick change from a slow ORR process to a quicker process. The E_onset_ can also be defined as the potential at which the current density reaches 5% of the diffusion-limited current (j_L_) or as the potential where the slope of the voltammogram surpasses 0.1 mA V^−1^ [59,60,61]. In the former approach, the plateau region of the curve corresponds to the limiting current, representing a stable current, typically resulting in mass transport limitations rather than kinetic control. This technique is commonly used since it relates the starting potential to a specific current flow, marking the beginning of the ORR process. The latter definition correlates the rate of current change with the transition between inactive and active reaction conditions. It focuses on the reaction kinetics of the ORR process rather than the current level.

These definitions offer valuable insights but highlight different aspects of the ORR process—current initiation vs. reaction kinetics. The inflection point method focuses on the sharpest change in the voltammogram, whereas the other methods mentioned set specific, externally defined criteria. They are all used to characterize the onset potential of the ORR but emphasize different aspects of the current–voltage relationship. When comparing results from different sources, it is essential to note which definition is used.

On the other hand, the half-wave potential is when the current density is at the halfway point of the diffusion-limited current density. The E_1/2_ evaluates the efficiency of the electrocatalyst in advancing ORRs. Usually, a more positive E_1/2_ signifies improved catalytic activity, implying that the ORR can occur at a higher potential, closer to the thermodynamic equilibrium potential of the reaction [61,62].

The fourth parameter is the electron transfer number (*n*), which assesses the reduction mechanism, i.e., either the 2e^−^/2H^+^ or 4e^−^/4H^+^ pathway. A few studies have shown that *n* depends on the angular velocity of a rotating electrode [63,64,65]. The n value depends on the angular velocity due to the mass transport of O_2_ to the electrode surface. The angular velocity influences how quickly O_2_ can diffuse to the surface of the electrode, which affects the diffusion layer. The diffusion layer becomes thinner at higher angular velocities, enhancing mass transport [66,67]. Now, there are two methods to evaluate n—RRDE and RDE.

The RRDE method uses a rotating disk electrode with a Pt or Au-based ring at a particular rotational speed to measure the *n* during the ORR process. When the electrocatalyst reduces the O_2_ on the disk section, centrifugal force drives the intermediate product to the ring section for oxidation. Take the ORR in acid electrolyte as an example; when oxygen is reduced via the 2e^−^ route in an acidic medium, it produces an H_2_O_2_ intermediate, which is subsequently oxidized to H_2_O on the ring. The electron transfer number can be determined by analyzing the recorded currents from both electrodes, as shown in Equation (8). The RRDE method can also quantify the H_2_O_2_ formation (Equation (9)) on the ring electrode, allowing for a more accurate n determination [68,69,70].

(8) n=4×IdId+IrN

(9) $$\%H_{2}O_{2}=200\times \frac{I_{r}}{N\times I_{d}+I_{r}}\;$$

where *I_d_* and *I_r_* are the disk current and ring currents, respectively, and *N* is the calibrated collection efficiency of the electrode.

The RDE method is based on the Koutecky–Levich (K-L) theory, which measures the electron transfer number using a series of rotational speeds, in general, between 400 and 2500 RPM. The theory describes the current density behavior on the rotating disk electrode using the equations below [71,72].

(10) $$\frac{1}{j}=\frac{1}{j_{L}}+\frac{1}{j_{K}}=\frac{1}{{B\omega }^{1/2}}+\frac{1}{nFkC_{O_{2}}}$$

(11) $$B=0.62nFC_{O_{2}}{D_{O_{2}}}^{2/3}\upsilon^{-1/6}$$

where *j* is the measured current density, *j_L_* is the diffusion-limited current density, and *j_K_* is the kinetic current density. *j_L_* is proportional to the square root of angular velocity (ω), and *k* is the electron transfer rate constant. F is the Faraday constant (96.485 kCmol^−1^); $C_{O_{2}}$ is the concentration of dissolved O_2_; $D_{O_{2}}$ is the diffusion coefficient of O_2_; and ν is the kinetic viscosity of the electrolyte. The *n* value is determined from the slope of the linear plot of *j*^−1^ versus $\omega^{1/2}$. However, The K-L theory makes two assumptions that make it unsuitable for ORR measurement. These assumptions are as follows: (1) the ORR is a single-step reaction with *n* being constant; (2) the surface of the electrode used for measurement is smooth, and the catalyst layer is thin [63,73,74].

Studies that have used RRDE and K-L theory measurements to obtain the *n* value reveal discrepancies between the results. For example, Xu, Qsynthesized three cobalt porphyrin composites (named CoPor/C, BTD-CoPor/C and TPA-BTD-CoPor/C) using carbon black for ORR measurements in an alkaline solution. When determining the *n* value, they found that the values derived from the RRDE method were 0.5–0.9 units greater than those obtained using the K-L theory method [75]. In another publication, Chen, Y. et al. created cobalt porphyrins using the doner-pi bridge-acceptor (D-π-A) design principle to assess ORR performance in 0.5 M H_2_SO_4_. The cobalt porphyrins, referred to as EGZ1/C, EGZ2/C and EGZ3/C, have *n* values of 2.7, 2.9, and 2.9, respectively, when measured with the RRDE method. When measured with the K-L theory, the composites have values closer to or lower than 2, i.e., 2.2 for EGZ1, 1.3 for EGZ2, and 1.8 for EGZ3 [76]. Both publications reasoned that the difference in values is caused by the surface roughness of the carbon black support material, which can dramatically change the measurement conditions, moving it well beyond the assumptions of the K-L model. Given that the ORR is a multi-electron pathway and the results in published work, it is safe to say the RRDE method is better suited for finding the electron transfer number as opposed to the RDE method.

### 2.2. ORR Mechanism

**Figure 3:**
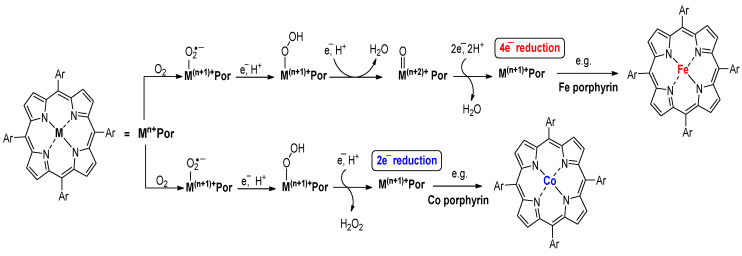
Proposed pathways of the ORR mechanism.

The ORR mechanism, shown in Figure 3, suggests that the selectivity of the ORR pathway depends on the transition metal coordinated within the porphyrin. As previously mentioned, iron porphyrins can catalyze the reduction of O_2_ to H_2_O via a four-electron process, in contrast with cobalt porphyrin, which tends to catalyze the reduction of O_2_ to H_2_O_2_ through the two-electron process [38].

**Figure 4:**
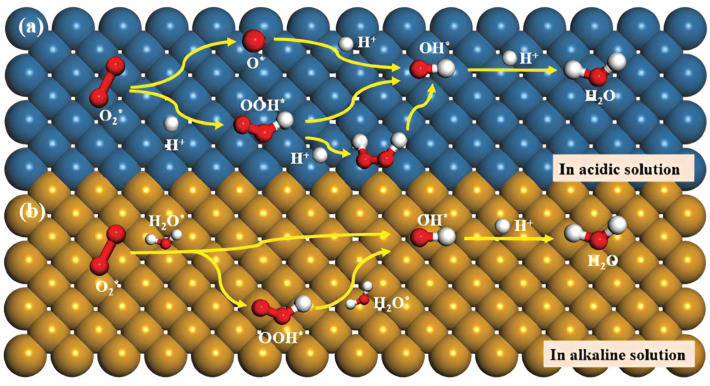
The schematic diagram of intermediates formed during the proposed pathways for ORR mechanisms in (**a**) acidic and (**b**) alkaline electrolytes. Reproduced from Ref. [77] with permission from Advanced Science, copyright 2023.

Figure 4 provides the intermediates that formed during the ORR process. It begins with the heterolytic cleavage of the O=O bond. The asterisk (*) denotes active catalyst sites.

O_2_* → O* + O* (12)

Breaking the molecular oxygen through heterolytic cleavage produces important metal–oxo intermediate species at the terminal. Iron porphyrins can catalyze O_2_ to H_2_O via the 4e^−^ reduction pathway under acidic conditions due to the facile formation of terminal metal–oxo intermediates. The 4e^−^ reduction pathway involves three primary intermediates: OOH*, O*, and OH*. The catalytic reaction mechanism primarily depends on the energy barrier of oxygen dissociation, mainly through couples of associative reactions, as shown below [77,78].

O_2_* + H^+^ + e^−^ → OOH* (13)

OOH* → O* + OH*(14)

O* + H^+^ + e^−^ → OH* (15)

OH* + H^+^ + e^−^ → H_2_O* (16)

According to the paths above, O_2_* combines with a proton and an electron to form OOH* (Equation (12)). Subsequently, the OOH* splits into O* and OH* by breaking the O=O bond (Equation (13)). The O* then reacts further with another proton and electron, forming OH* (Equation (14)). Lastly, the OH* is reduced to water by gaining a proton and an electron (Equation (15)). The intermediate OOH*, O*, and OH* are vital in the ORR process, and the adsorption energy with which they bind to the metal active sites determines the efficiency and selectivity of the ORR [77,79,80].

The four-electron route can be achieved in an alkaline medium when O_2_* directly interacts with water and an electron. This reaction produces the intermediate OH* through a single, continuous four-electron process, shown below [77].

O_2_* + 2H_2_O* + 4e^−^ → 4OH* (17)

**Figure 5:**
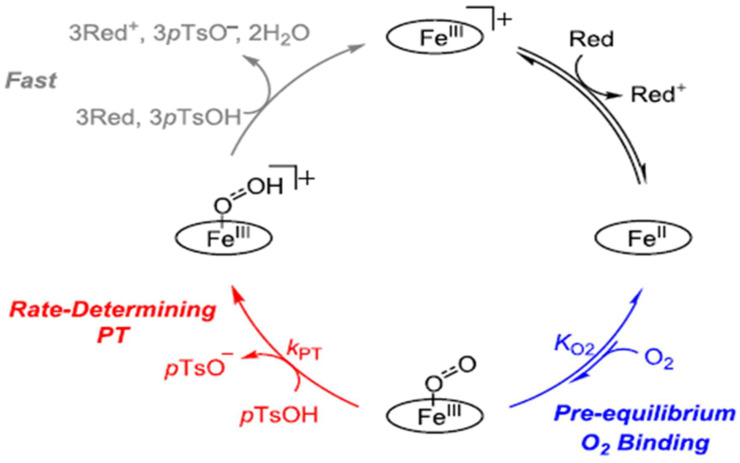
Proposed mechanism for oxygen reduction catalyzed by Fe^II^TPP. Reproduced from Ref. [81] with permission from Journal of the American Chemical Society, copyright 2019.

On the other hand, Pegis, M.L. studied the reduction mechanism catalyzed by iron (II) tetraphenylporphyrins (Fe^II^TPP) in N,N′-dimethylformamide using decamethyl-ferrocene as a soluble reductant and para-toluenesulfonic acid (pTsOH) as the proton source. They used density functional theory (DFT) calculation, kinetic modeling, cyclic voltammetry, and other electrochemical and spectroscopic experiments to support the mechanism, as shown in Figure 5. In this mechanism, the electrode loaded with the Fe^II^TPP reduces [Fe^III^TPP]OTf to Fe^II^TPP, which then reversibly binds with O_2_ to form Fe^III^TPP(O_2_^●−^)—a ferric superoxide. The following stage involves the protonation of the ferric superoxide to create a perhydroxyl–iron(III) complex, [Fe^III^TPP(O_2_H^●^)]^+^. This step determines the rate of the reaction. Subsequently, this complex undergoes rapid reduction and protonation, generating two units of H_2_O and resetting the catalytic cycle [81].

**Figure 6:**
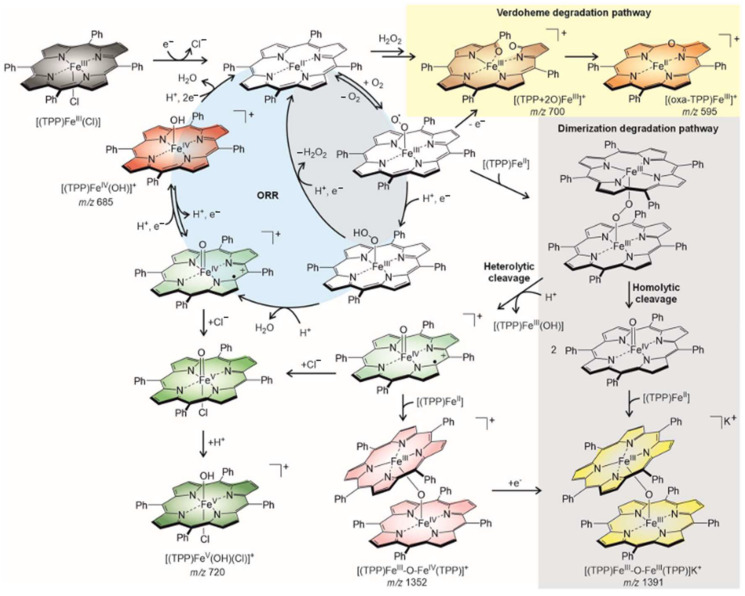
The electrocatalytic oxygen reduction reaction mechanism catalyzed by [Fe^II^TPP]. Reproduced from Ref. [82], free-to-use rights.

Surendran, A.K. et al. investigated the ORR mechanism catalyzed by an iron (II) tetraphenyl porphyrin complex [Fe^II^TPP]. They used voltammetry, electrochemical impedance spectroscopy, and electrospray ionization mass spectrometry to monitor the ORR process and track reaction intermediates. The entire mechanism can be seen in Figure 6. The mechanism commences with the reduction of [Fe^III^ClTPP)] to the [Fe^II^TPP] complex, where it converts O_2_ to two molecules of water through a series of proton and electron transfers. As the ORR process occurs, several intermediates are formed, along with several degradation pathways. The kinetics of the mechanism, as well as the number of intermediates created, are dependent on the proton concentrations [82].

## 3. Synthesis and Characterization of Iron Porphyrin-Based Composites

### 3.1. Synthesis

**Figure 7:**
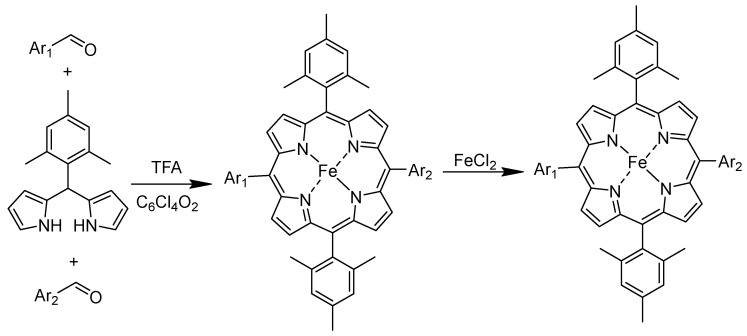
General synthetic route for iron porphyrins.

The synthesis of iron porphyrin-based components involves creating complexes where iron is coordinated with a porphyrin ring to facilitate electrocatalytic activity. Porphyrin is a heterocyclic macrocycle compound derived from four modified pyrroline subunits interconnected through their α-carbon atoms and methine bridges (=CH-), forming a large ring-like structure [83]. The porphyrin rings are first synthesized with various substituents, which allows for customizing their electronic and steric properties to suit specific applications. The porphyrin ring is typically made from the building blocks of pyrrole and an aldehyde (e.g., benzaldehyde or formaldehyde). The two are mixed in a solvent, such as dichloromethane, and undergo condensation for several hours, often using an acid catalyst (such as trifluoroacetic acid (TFA)). The protoporphyrin intermediate formed is oxidized (e.g., with tetrachlorobenzoquinone in the presence of air) to form the conjugated porphyrin ring fully [84]. The free-base porphyrin is isolated and purified, usually by column chromatography using an appropriate solvent system [85,86]. The synthesized porphyrin is then reacted with an iron salt (such as Fe^II^Cl_2_) to introduce the iron (II) ion into the center of the porphyrin ring. The metalation process involves using a solvent under reflux conditions to ensure complete metalation. The resulting iron porphyrin complex is purified through column chromatography [87,88,89]. Figure 7 shows a synthetic route for iron porphyrins.

In a nutshell, this process involves dissolving porphyrin in a solvent like dichloromethane, adding iron chloride (Fe^II^_2_ or Fe^III^Cl_3_) to the solution, heating the mixture under reflux for a few hours, and purifying the iron porphyrin complex by column chromatography. This direct metalation using iron salts is straightforward and efficient for incorporating iron into the porphyrin ring.

The formed iron porphyrin complex is usually combined with supporting materials such as carbon nanotubes, graphene, carbon black, or metal–organic frameworks (MOFs) to enhance electrical properties. Combining iron porphyrins with conductive materials significantly enhances their conductivity, catalytic activity, and stability. The composite is produced mainly through physical mixing, where the support material adsorbs the iron porphyrin. Usually, the iron porphyrin and the support material are ultrasonicated in a solvent to ensure even dispersion, and the solvent is evaporated to obtain the iron porphyrin-based composite material [90].

### 3.2. Characterization

**Figure 8:**
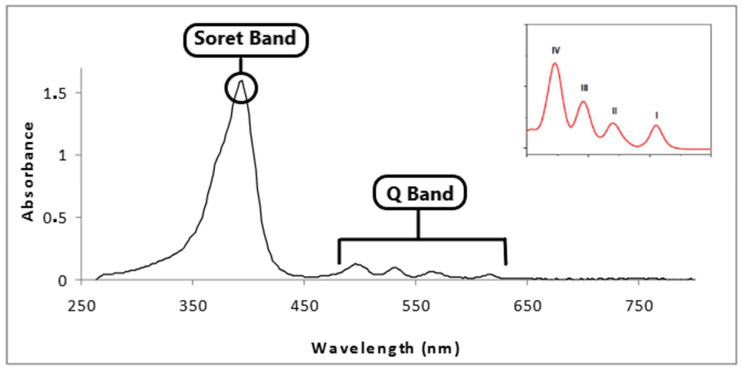
The UV-Vis spectrum of free-base porphyrin with an enlargement of Q band region. Reproduced from Ref. [93], free-to-use rights.

Characterizing the complex using advanced spectroscopic techniques is crucial for understanding iron porphyrins’ electronic structure and enhancing its ORR performance. Characterization methods such as proton/carbon nuclear magnetic resonance spectroscopy (H-NMR/C-NMR), X-ray photoelectron spectroscopy (XPS) and ultraviolet-visible spectroscopy (UV-vis) are typically used to investigate the structural information [77]. Proton NMR is used to analyze the chemical environment of hydrogen atoms in the porphyrin ring and those in the substituents attached to its peripheral meso-positions. Carbon NMR helps identify and confirm the carbon framework of the porphyrin rings. The porphyrin macrocycle comprises distinct carbon types—meso carbons, β-pyrrolic carbons, and the carbons of substituent groups [91]. XPS provide information on the electronic status of the iron center as well as the elemental composition of the complex and its valence state. UV/Vis spectroscopy has two characteristic absorption peaks—one at approximately 400–450 nm, known as the Soret band, and the other at approximately 500–800 nm, called the Q-band, as seen in Figure 8 [44,59,92,93]. The conjugated system’s π-π* transitions (electron transitions between molecular orbitals) give rise to this incredibly intense absorption of the Soret band. Additionally, it has 2–4 Q bands, commonly discernible between 500 and 750 nm. These Q absorption bands exhibit weaker absorption intensities because they originate from lower-energy transitions than the Soret band. It is usually broken into numerous peaks due to the differing energy levels available within the porphyrin structure, for instance, two bands for metalloporphyrins and four bands for the free-base catalog. FTIR spectroscopy is used to identify functional groups on the porphyrin ring. These functional groups are characterized by their vibrational mode, whether stretching, bending, etc., at different wavenumbers. It can provide information on molecular bonding and structure.

## 4. Effects of Iron Porphyrin Structure on ORR

The iron porphyrin structure has unique electronic properties that play a significant role in the ORR process [52]. The structure impacts the catalytic active site required for the adsorption of molecular oxygen and facilitates electron transfers during the ORR process. Lowering the overpotentials needed for ORRs can be achieved by fine-tuning the electronic status of the complex by tailoring the substituents at the peripheral positions or the functional groups attached [40,94]. A comprehensive understanding of how the molecular structure influences the ORR process leads to a better structure that reduces overpotential and improves ORR performance.

### 4.1. Axial Ligand Effects

The presence and nature of axial ligands bounded to the center iron atom can modulate the electronic environment of the complex. These ligands increase the electron density on the central ion, modifying the binding strength and activation of O_2_, influencing the ORR efficiency and selectivity. The increased electron density on the metal ion can facilitate electron transfer to the O_2,_ which lowers the activation energy for O=O cleavage [42,95]. Also, ligands affect the coordination number of the iron center, which restricts the available sites for oxygen binding and reduction. A lower coordination number provides more open sites for oxygen binding, which enhances ORR activity. The axial ligand interactions with the iron porphyrin can often improve ORR selectivity and efficiency [96,97].

**Figure 9:**
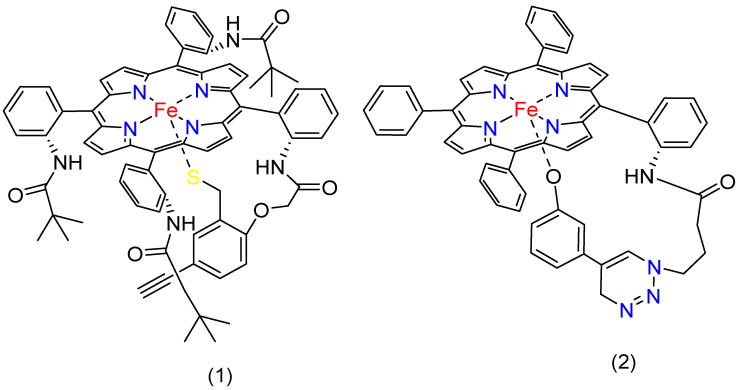
Molecular structure of complexes **1**–**2**.

Numerous publications have demonstrated the vital role of those axial ligands on the iron porphyrin complexes. Complexes **1** and **2** (Figure 9) are two iron porphyrins linked with anionic ligands—one with a thiolate ligand and the other with a phenolate ligand. The complexes were physisorbed onto graphite surfaces and then covalently attached to an azide-terminated SAM (Self-Assembled Monolayer) on Au electrodes. They were assessed in a pH 7 buffer solution of 0.1 M KPF_6_, with Pt as the counter electrode and Ag/AgCl as the reference electrode. According to the analysis, the catalysts could selectively reduce O_2_ to H_2_O via the 4e^−^/4H^+^ route at a remarkable rate [98].

**Figure 10:**
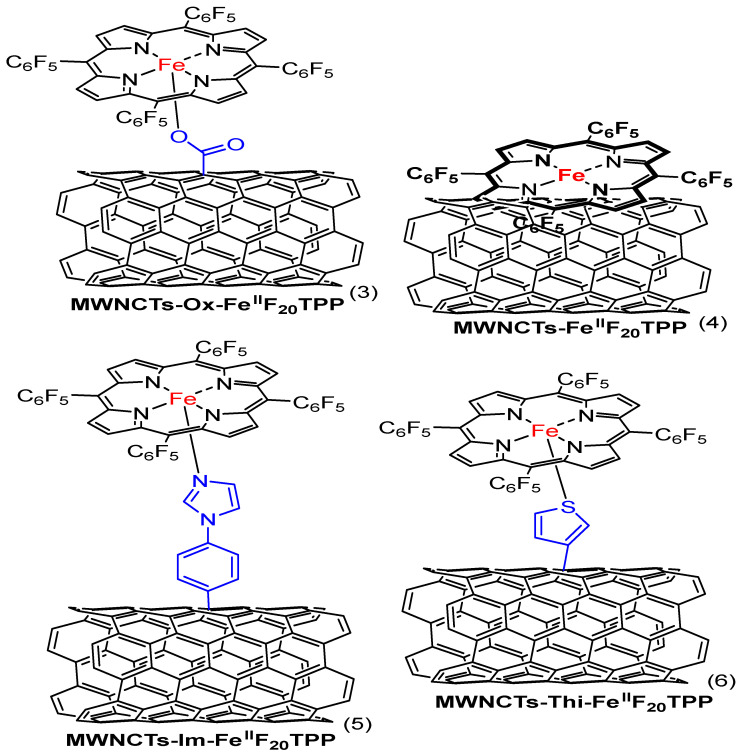
Molecular structure of complexes **3**–**6**.

Another report shows that researchers created a composite by attaching the catalysts to multi-walled carbon nanotubes (MWCNTs). On the surface of the nanotubes, the metal-based catalysts (complexes **3**–**6**, Figure 10) are coupled with iron porphyrin (Fe^II^F_20_TPP) with thiophene, imidazole, and carboxylate axial ligands. The study demonstrated that the axial ligands coordinated with the iron porphyrin resulted in an increased oxygen reduction rate and an improved 4-electron transfer efficiency. The composite catalyst with imidazole coordination (MWCNTs-Im-Fe^II^F_20_TPP) showed the most optimistic E_onset_ (1.04 V vs. RHE) and E_1/2_ (0.87 V vs. RHE) among the synthesized catalysts. It also has an electron transfer number value of nearly 4. Table 1 presents the catalytic performance of iron porphyrin electrocatalysts for ORRs. Furthermore, when they evaluated the performance of MWCNTs-Im-Fe^II^F_20_TPP towards ORRs in a zinc battery, it showed the most effective activity, comparable to Pt/C catalysts [47].

**Figure 11:**
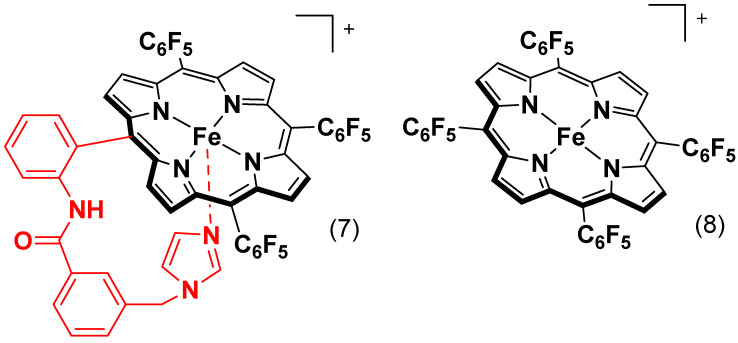
Molecular structure of complexes **7**–**8**.

Xie, L. et al. synthesized two iron porphyrin complexes inspired by enzymes. One of the complexes bears an imidazole ligand (complex **7**, Figure 11), while the other is imidazole-free (complex **8**, Figure 11), with both supported on carbon nanotubes (CNTs). The tethered imidazole ligand increased the electron density on the iron core, leading to improved O_2_ binding and O=O bond cleaving. As a result, the iron complex with the tethered imidazole showed better ORR performance compared with the free imidazole analog. The E_onset_ value for 1/CNT is 930 mV and an E_1/2_ value of 840 mV, while 2/CNT has E_onset_ and E_1/2_ values of 850 mV and 680 mV, respectively. Both catalysts showed an electron transfer number value near 4. Furthermore, the imidazole iron porphyrin was used to construct a Zn-battery, which exhibited performance competitive to Pt/Ir-based materials [57].

The participation of axial ligands cannot be overestimated regarding the changes in the catalytic properties of iron porphyrin complexes during the ORR process. Several review articles emphasize axial ligands’ effectiveness on iron porphyrins in improving ORR activity [52,97]. Our findings also highlight the usefulness of axial ligands bound to the iron center in porphyrin complexes. They show the role of electron density modulation by the ligands at the iron center for electron transfer to oxygen, effectively lowering the activation energy towards the O=O bond cleavage. Their ability to retain high electron density at the metal center reduces the activation energy and improves the efficiency of the ORR. The adjustment increases the selectivity towards the desired 4e^−^ reduction pathway to water and minimizes overpotentials significantly.

Despite such advances, there are several limitations to using axial ligands in ORR catalysis. While thiolate, phenolate, and imidazole enhance the electron density at the iron center, which is favorable for reducing the activation energy and enhancing electron transfer, these ligands may introduce some problems regarding catalyst stability. Under acidic conditions, ligand dissociation or degradation may occur, leading to a sharp decline in catalytic efficiency with time [104]. The catalyst’s durability can suffer under operational conditions if the ligands are easily displaced or degraded. One of the major challenges of ligand design thus concerns the construction of molecules displaying enhanced catalytic activity and structural stability under extreme conditions. While a higher electron density at the active metal center generally leads to better ORR performance, an electron density that is too high might also promote the formation of unwanted by-products or reactivity that compete with the desired ORR pathway [105]. Therefore, when selecting axial ligands, strategies that promote the proper balancing of electron distribution for optimization while decreasing unfavorable intermediate formation must be employed to develop future catalysts that can optimize activity and stability.

As mentioned, the coordination number of the iron center is another crucial factor in changing axial ligands. Lower coordination numbers offer more free sites for oxygen binding, increasing ORR activity. On the other hand, lower coordination can destabilize the catalyst because it becomes more vulnerable to unfavorable conditions such as high voltages or reactive intermediates. It would be ideal to reach that delicate balance between leaving enough open coordination sites to achieve suitable ORR activities and maintaining the structural integrity of the complex. This challenge will probably be understood using multi-functional ligands or even those capable of forming stable bonds under different operational conditions. Future efforts should be directed toward ligand syntheses that will result in even better catalytic performance and catalyst durability under harsh operational conditions. Solving the instability issues noted above and further electronic optimization of the ligands’ properties may allow for a class of genuinely efficient and commercially feasible iron porphyrin-based catalysts that will become paramount for developing green energy technologies.

### 4.2. Substituents Effect

Modifications to the peripheral substituents at the meso- or β-positions on the porphyrin ring can influence the electron density at the iron center [86,106]. Tailoring at the meso-positions is often favored over the β-positions for several reasons. First, alterations at the meso-positions of porphyrins are significantly more straightforward than the β-positions. Meso-substituted porphyrins can be produced using several established techniques, including the reaction of pyrrole with aromatic aldehydes, providing greater versatility and improved yields. β-substituted porphyrins have more complicated synthetic routes, which results in a lower yield [107]. Second, substituents at the meso-positions tend to exert greater influence on the electronic properties of the porphyrin structure. Research indicates that the meso-substituents can change the redox potential of porphyrins more effectively than β-substituents, which is vital in catalysis applications [108,109]. Third, porphyrins altered at the meso-site normally maintain their planar structure better than those adjusted at the β-site. Changes at the meso-position do not disrupt the ring’s core conjugation and do not produce major steric hindrances, which allows for the retention of the flat form. Adjustments at the β-positions can frequently cause steric problems, disrupting the ring’s planarity. This can negatively affect the porphyrin’s electronic properties and even hinder the accessibility of reactants to the catalytic site [110,111].

Substituent alteration in the peripheral positions offers a versatile method of tuning the electronic and steric properties of the catalyst, thus providing a means to fine-tune both selectivity and efficiency [86]. Electron-donating or -withdrawing groups alter the redox potential of the iron porphyrin complex, thereby affecting the ORR kinetics and pathways. Studies indicate that electron-withdrawing groups tend to enhance the ORR activity, while electron-donating groups help stabilize the vital intermediate in the 4e^−^ pathway [97,112,113].

**Figure 12:**
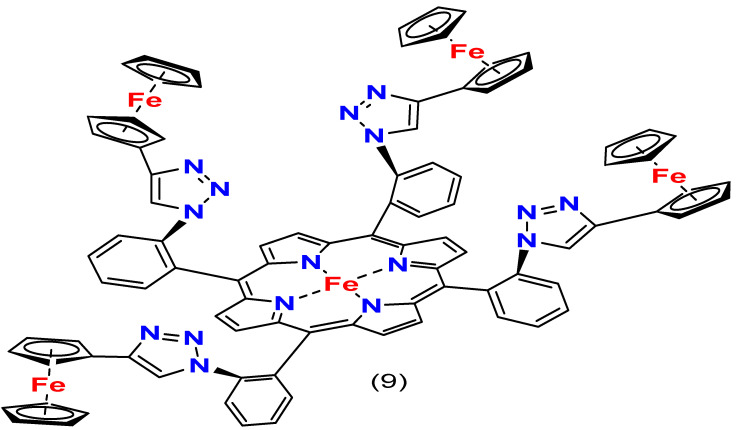
Molecular structure of complex **9**.

Many studies reveal the effectiveness of modifying the substituents on the iron porphyrin ring. For instance, Mittra, K. et al. found that an iron porphyrin catalyst with four ferrocenes and a hydrogen bonding distal pocket (complexes **9**, Figure 12) promotes the reduction of O_2_ through the 4e^−^/4H^+^ process in an organic solvent under homogenous conditions in the presence of trifluoromethanesulphonic acid. This catalyst exhibits consistent efficiency and selectivity over a range of pH values, and it can perform the same oxygen reduction in an aqueous solution under heterogeneous conditions [99].

**Figure 13:**
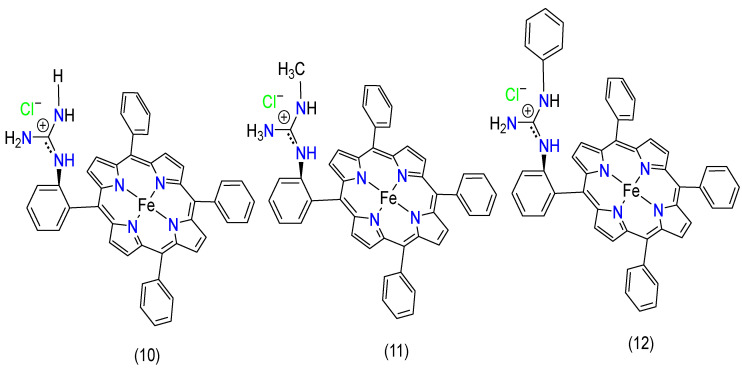
Molecular structure of complexes **10**–**12**.

In 2019, Ghatak, A. et al. formulated iron porphyrins, attaching pendant guanidine groups by covalent bonds, resulting in complexes **10**–**12** (Figure 13). Their findings indicate improved kinetics and selectivity for the 4e^−^/4H^+^ pathway during ORRs. The researchers also introduced an axial imidazole ligand to increase selectivity, which produced minimal partially reduced oxygen species (PROS) during the ORR process [100].

**Figure 14:**
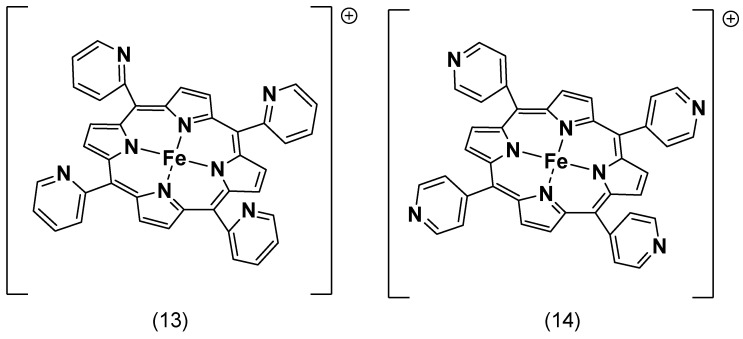
Molecular structure of complexes **13**–**14**.

Similarly, two iron porphyrin complexes with pyridine substituents (complexes **13** and **14**, Figure 14) were synthesized by Matson, B.D. et al. for ORRs in an acid electrolyte, which showed selectivity towards the 4e^−^/4H^+^ reduction [101]. Lü, A. et al. synthesized five variations of iron tetraphenylporphyrins with different electron-influencing groups on the phenyl ring and tested them for ORR activity in an acidic solution. The study found that iron porphyrins with electron-withdrawing substituents demonstrated higher efficiency in catalyzing oxygen reduction than those with electron-donating substituents [114].

According to the reported studies, tailoring appropriate substituents at the meso-position of the iron porphyrin ring has enhanced the ability to reduce activation energy and improve the catalyst’s capability to promote the 4e^−^ route. Although the results mentioned above amply illustrate the gain of meso-substitution on the catalytic efficiency of iron porphyrins, the perspective of further review papers discussing more significant trends is essential. For example, Banham et al. (2015) review non-precious metal catalysts’ stability and durability for the oxygen reduction reaction [2]. They noted that though electron-withdrawing groups at the meso-positions can improve ORR activity through increased redox potential and improved electron transfer, these gains must be carefully weighed with stability under realistic operating conditions. In real applications, the catalysts are usually exposed to harsh acidic or alkaline conditions that trigger degradation. According to Banham et al., further stabilization modifications significantly mitigate such degradation pathways. This becomes more cardinal for the longevity of the catalyst in fuel cell environments, where stability over a long period is as important as activity.

This goes nicely in line with our discussion above about meso-substitution. While the meso-substituents successfully tune the redox potential and increase catalytic efficiencies, one should also pay attention to retaining catalyst durability.

Another review, that of Jaouen et al. (2011), about the non-precious metal catalysis of ORRs, underlined iron porphyrin as a promising electrocatalyst [115]. It showed attention to peripheral substituents because of their ability to tune the electronic properties of the catalyst finely; for example, electron-donating groups at the meso-position efficiently enhance the electron density at the iron center—a crucial issue in keeping high catalytic activity. Moreover, Jaouen et al. discussed that the electronic stabilization of reaction intermediates could decrease possible side products like H_2_O_2_ formations. Indeed, by using an iron–oxygen complex in a stable state throughout the ORR process, those side reactions that otherwise occur and result in reduced efficiency and selectivity might be minimal.

These results pointed out the effects of substituent selection at the meso-positions of the iron porphyrin ring. Indeed, the intentional incorporation of electron-withdrawing or -donating groups at these positions provides a potent method for tuning the redox properties of the catalyst, thus offering further control of the pathways of ORRs and increasing efficiency. Works such as Banham et al. (2015) and Jaouen et al. (2011) caution that while catalytic efficiency is being developed via meso-substitution, there should be a corresponding impetus toward improvement in the stability of the catalyst under operation. Combining meso-substitution with other modification methodologies might eventually pave the way for realizing an active yet inexpensive iron porphyrin-based catalyst in energy conversion technologies.

Subsequent work should focus on the strategic positioning of acceptor and donor moieties to maximize the electron transfer rate while addressing stability concerns. Also, integrating meso-substituted iron porphyrin catalysts with other strategies, such as axial ligand coordination, metal–metal cooperation, or within a metal–organic framework (MOF), should be promising approaches [116,117,118]. These combinations could enable concurrent optimizations of activities, selectivity, and stabilities toward practical fuel cell applications. Integrating various modifications can comprehensively boost the catalytic activity and long-term stability of iron porphyrin-based catalysts, bringing these electrocatalysts closer to realizing practical and cost-effective alternatives to platinum-based systems for energy conversion technologies.

### 4.3. Functional Group Effect

Functional groups attached to the porphyrin ring can effectively modify the ORR activity [27]. The functional groups can participate in secondary interactions with reactants or intermediates, potentially stabilizing transition states during ORRs [119]. Moreover, functional groups facilitating proton transfer to and from the active site can significantly enhance ORR performance [120,121]. Various functional groups such as carboxyl, methyl, and amino groups have been shown to lower the activation energy for proton-coupled transfer steps, a critical aspect of the ORR mechanism. Conversely, the absence of functional groups may result in the formation of harmful species such as H_2_O_2_ [122,123]. Therefore, the selection and positioning of functional groups on the porphyrin structure are fundamental to increasing the stability and selectivity of iron porphyrins in ORR applications.

**Figure 15:**
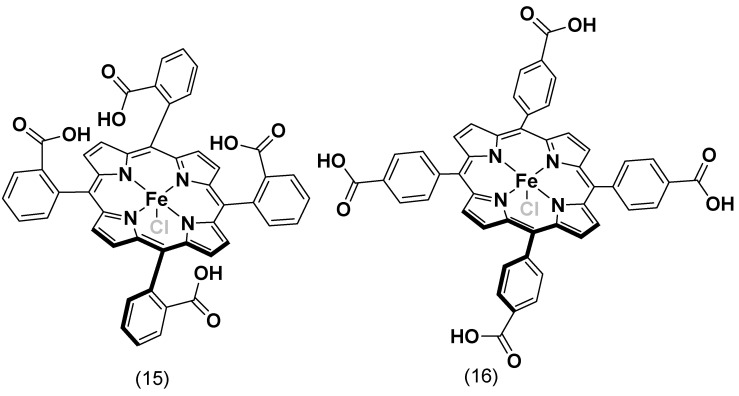
Molecular structure of complexes **15**–**16**.

Studies, like the one conducted by Carver, C.T. et al., show the viability of using functional groups to enhance the ORR performance of iron porphyrins. The researchers synthesized two iron porphyrins with carboxyphenyl groups pointing to or away from the center ion (complexes **15** and **16**, Figure 15). The carboxyphenyl groups pointed towards the center can perform proton relays, while the others have distal relays. When the ORR performance was evaluated, molecule **15** showed selectivity towards the 4e^−^/4H^+^ pathway, while molecule **16** formed substantial H_2_O_2_. Therefore, functional groups that can deliver protons can substantially enhance the selectivity of ORRs [124].

**Figure 16:**
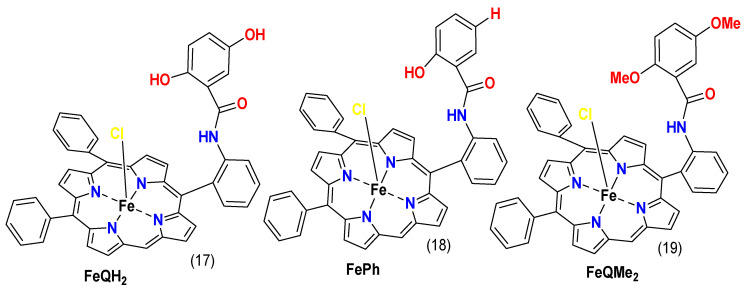
Molecular structure of complexes **17**–**19**.

**Figure 17:**
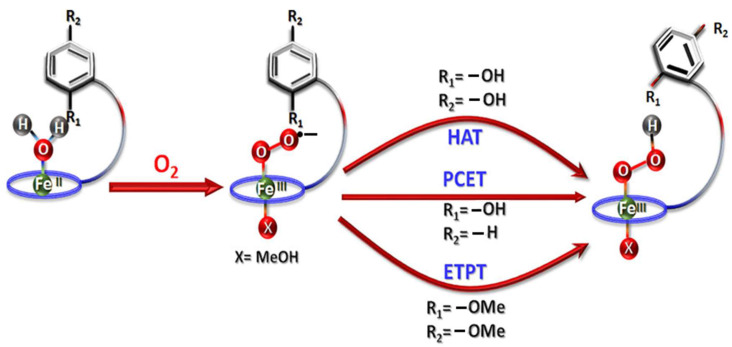
PCET and HAT mechanism during ORR. Reproduced from Ref. [102] with permission from Advanced Science, copyright 2020.

Complexes **17**–**19** (Figure 16), which bear covalently coupled pendant phenol, quinol, and methyl-quinone, were studied by Singha, A. et al. (2020). The complexes were physiadsorbed on an edge-plane graphite (EPG) electrode and assessed for ORR activity in a 0.1 M KPF_6_ phosphate-buffer solution. These composites converted O_2_ to H_2_O through the 4e^−^/4H^+^ pathway, according to RDE data. Furthermore, density functional theory calculations and electrochemical results reveal that in the presence of the pendant phenol, the Fe^III^–O_2_ species reduction to Fe^III^–OOH occurs through a proton-coupled electron transfer (PCET) mechanism. However, with a pendant quinol, the process follows a hydrogen atom transfer (HAT) route. Without the hydroxyl group, the reduction in molecular oxygen involves an initial electron transfer, followed by a proton transfer [102]. The mechanism can be seen in Figure 17.

**Figure 18:**
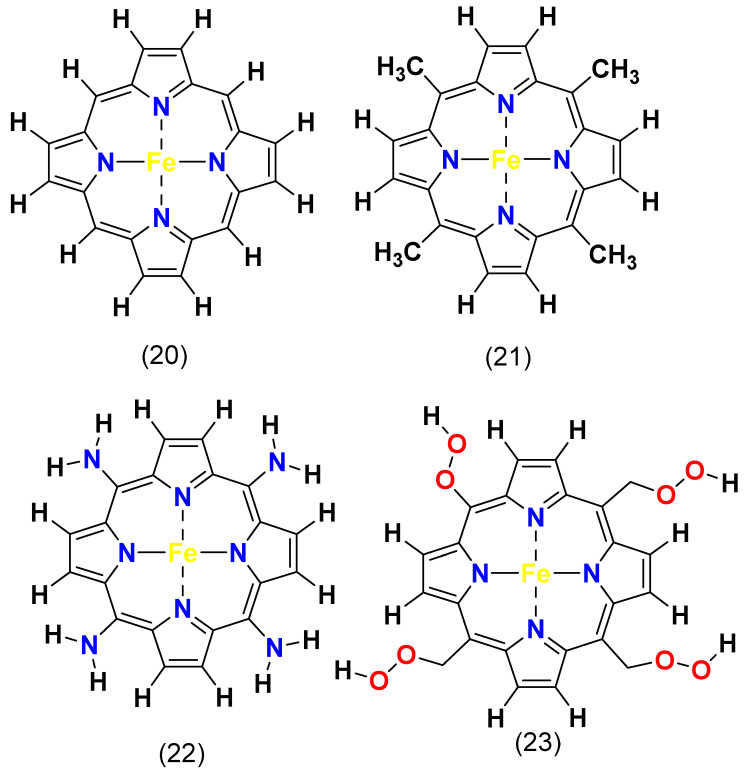
Molecular structure of complexes **20**–**23**.

In their study, Dung, T.P. et al. examined the impact of carboxyl (–COOH), methyl (–CH_3_), and amino (–NH_2_) functional groups at the meso-position of the iron porphyrin. Figure 18 illustrates the structure of the iron porphyrin (Fe^II^Por) complex **20**, meso-tetramethyl iron porphyrin (Fe^II^TMP, porphyrin–(CH_3_)_4_) complex **21**, meso-tetraamino iron porphyrin (Fe^II^TAP, porphyrin–(NH_2_)_4_) complex **22**, and meso-tetracarboxylic iron porphyrin (Fe^II^TCP, porphyrin–(COOH)_4_) complex **23**. Through Van der Waals density functional theory (Vdw-DFT) calculations and thermodynamic modeling, they determined that iron porphyrins substituted with methyl (–CH_3_) and amino (–NH_2_) groups were the most effective functional groups. These substituents lowered the activation energy required for ORRs, thereby boosting the catalytic activity for ORRs [123].

These studies have shown that functional groups can significantly improve catalyst performance, mainly through the electron/proton-coupled transfer processes. Functional groups can modulate the local electronic structure around the iron center, thereby influencing the binding energies of intermediates such as OOH*, O*, and OH*, and improving the catalytic selectivity and efficiency. Functional groups with proton-donating or proton-accepting capabilities, such as carboxyl or amino groups, can create a favorable microenvironment that stabilizes the reaction intermediates through hydrogen bonding [123]. This secondary coordination sphere effect mimics natural enzyme behavior and can facilitate proton transfer, further accelerating the ORR process [78]. Introducing such groups provides a promising route to overcome limitations set by traditional scaling relationships, offering new avenues for enhancing ORR performance on iron porphyrin catalysts.

While our findings indicate that functional groups influence ORR performance, challenges remain. The most critical challenge in functionalized catalysts involves their stability when considering operational situations. The carboxyl or amino functional groups that provide excellent ORRs will most likely undergo some form of chemical degradation, especially under the highly oxidative conditions expected in the atmosphere where the ORR functions [125]. Consequently, further research should focus on elucidating functional groups that would provide the requisite catalytic activity and enhance the catalyst’s stability and durability. The design and construction of more robust functionalized iron porphyrin complexes, probably incorporating electronically stable substituents, will enhance activity and lifetime in such catalysts.

Another challenge involves the optimization of functional group placement on the porphyrin ring. From the difference in properties of complexes **15** and **16**, one can easily illustrate that the orientation of the functional groups plays a vital role in defining catalytic performance. Generally, functional groups suited to support efficient proton relays are better at enhancing selectivity towards complete reduction [121,122]. At the same time, ill-positioned functional groups may form partial reduction products such as H_2_O_2_ through various side reactions [126,127]. Careful attention to functional group orientation must be informed by computational modeling and mechanistic studies to drive catalyst design for maximum efficiency and selectivity.

### 4.4. Iron–Metal Coupled Porphyrins

Another approach to enhance iron porphyrins’ catalytic activity and stability involves introducing an additional transition metal ion. The interaction between two transition metals can modify the electronic structure of the porphyrins through synergistic effects, ultimately leading to improved ORR performance compared to single-metal counterparts [128]. The additional metal can contribute or withdraw electron density from the iron center, thereby adjusting the Fe^II^-based active sites to enable more effective O_2_ adsorption and reduction [129,130,131]. The adsorbed O_2_ has the potential to bind to both metal centers, forming an M_1_–O–O–M_2_ species. This lowers the activation energy needed to break the O=O bond, further enhancing catalytic efficiency [27]. Designing the bimetallic system would involve fine-tuning the distance between metal centers, coordination environment, and oxidation state to ensure optimal interaction between the metals and the substrate [132]. Additionally, there would be multiple active sites where different steps of the ORR process can occur. For instance, while one metal aids in the adsorption of O_2_, the other could drive the proton-coupled electron transfer process, thereby promoting the 4e^−^ reduction pathway [133].

The catalytic performance of Fe^III^TPyPCl, an iron porphyrin covalently linked to a pyridine group, was significantly improved by Maruyama, J. et al. through the incorporation of various transition metal ions (Co^2+^, Ni^2+^, Cu^2+^). This enhancement is attributed to the coordination of these metal ions by the pyridyl-N group attached to the porphyrin at the meso-position, resulting in electronic interactions between the iron center and the additional metal ions. These interactions, facilitated by coordination and conjugated bonds within the porphyrin structure, enhance the ORR activity of the porphyrin, emphasizing the importance of introducing transition metal ions [134].

**Figure 19:**
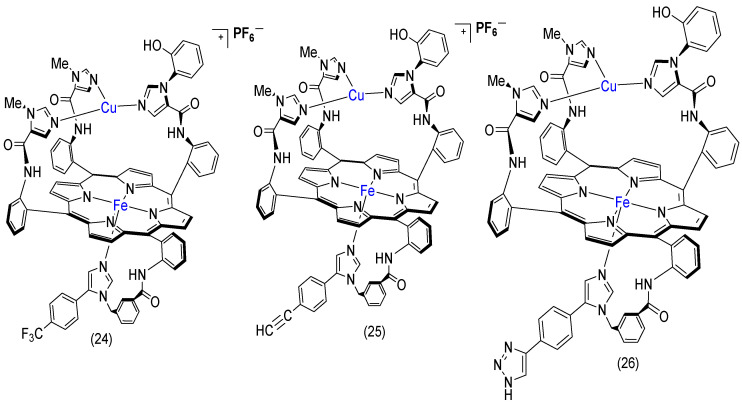
Molecular structure of complexes **24**–**26**.

Collman, J.P. et al. have reported several cytochrome c oxidase (CcO) models of bimetallic Fe-Cu complexes (Figure 19). These models can catalyze the 4e^−^ reduction of O_2_ to H_2_O [51,130,135,136].

**Figure 20:**
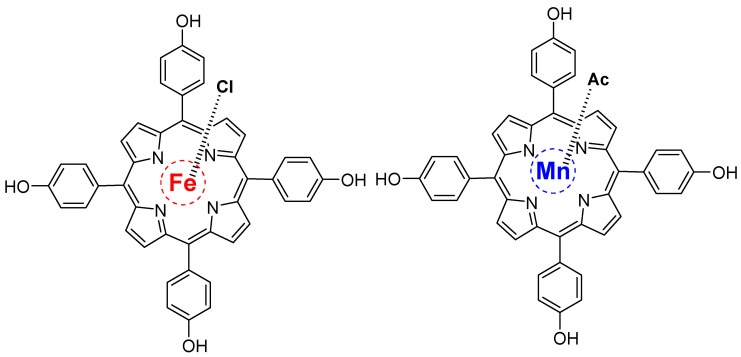
Iron (III) and manganese (III) porphyrins used to construct the bimetallic composite.

Kuzmin, S.M. et al. investigated the synergistic effects of iron (III) and manganese (III) porphyrins in a polymer catalyst for oxygen electroreduction. They employed superoxide-assisted electrochemical deposition to produce films of individual metalloporphyrins and bimetallic composites. Figure 20 shows the molecular structure of individual metalloporphyrins combined to form the bimetallic composite. The results show that the bimetallic composite film exhibits lower overpotentials than individual porphyrins. Hence, catalytic activity and energy characteristics are improved because of the synergistic effects [137].

**Figure 21:**
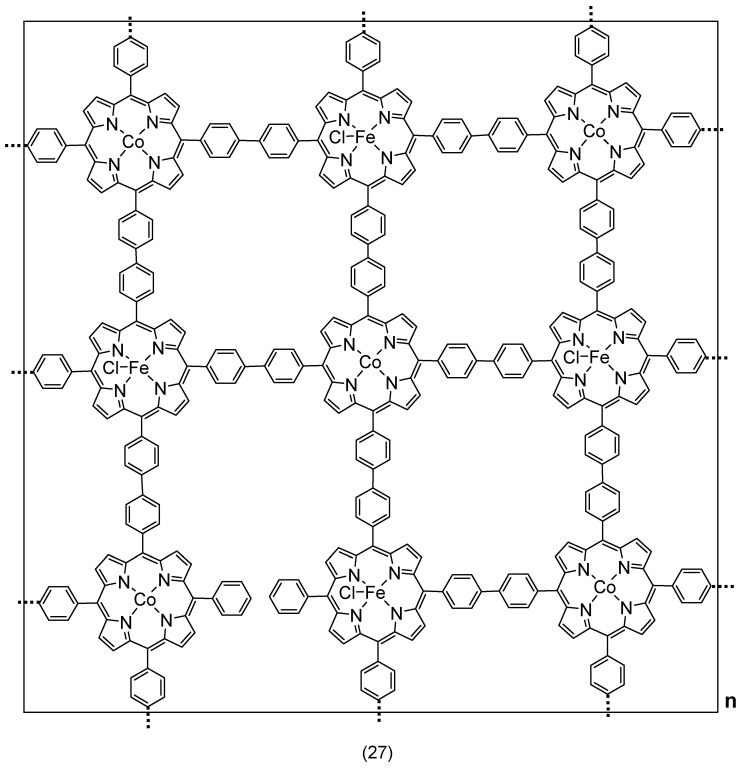
Molecular structure of complex **27**.

Brüller, S. et al. synthesized complex **27** (Figure 21) using Suzuki cross-coupling polycondensation to investigate the ORR performance of bimetallic Fe-Co porphyrin polymer catalysts. Their research demonstrates high selectivity, stability, and benefits of combining iron (III) and cobalt (II) active sites. The results show that the bimetallic catalyst exhibited the highest catalytic activity towards ORRs because of the synergistic effect for cobalt and iron active sites, achieving an E_onset_ = 0.88 V and E_1/2_ = 0.78 V, which is only 0.06 V less than the Pt/C catalyst. Furthermore, the bimetallic catalyst is exclusively selective to the 4e^−^ transfer for reducing O_2_ to H_2_O [103].

These iron–metal coupled systems have led to noteworthy enhancements in the electrocatalyst’s catalytic activity, selectivity, and stability. As mentioned before, the multiple reactivity sites are advantageous because each metal can facilitate different stages in the ORR process and enable the desired transfer of 4-electrons. Due to the presence of multiple metal centers, the catalytic mechanism is more flexible, allowing the catalysts to change their characteristics with respect to reaction conditions, which is a very effective way to maintain high efficiency and selectivity for a long time. However, despite these promising improvements achieved with iron–metal coupled systems, several challenges must be overcome before their full deployment can be realized. The one critical problem pertains to the stability of these bimetallic complexes under operational conditions. The presence of two different metals within the same complex introduces galvanic corrosion, whereby one metal corrodes preferentially in the presence of the other [138]. That could be one reason for the deterioration in the catalytic structure and decreased activity over time. Future efforts must be focused on optimizing the coordination environment and fitting oxidation states for both metals, which must be stable under the conditions of ORRs.

Other challenges include optimization issues regarding the distance between the metal centers. There should be optimal distances between metals for the desired synergy. If too far, it may cancel the synergistic effects; if too close, steric hindrance could be detrimental to the adsorption of reactants and the general catalytic process [128,139]. The design of bimetallic catalysts requires an optimal distance to realize a maximum cooperative effect with a minimum steric interference.

Future efforts should optimize the coordination environment, oxidation states, and intermetallic distances to maximize the synergy between the two metals’ active centers and catalytic performance and stability. Provided these challenges are appropriately met, iron–metal coupled porphyrins may eventually emerge as quite active and competitive systems compared to platinum-based catalysts in energy conversion applications, particularly fuel cells.

### 4.5. Iron Porphyrin-Based Metal–Organic Framework

Metal–organic frameworks consist of metal ions or clusters bonded to organic ligands, creating a repetitive three-dimensional structure with a large surface area and adjustable pore size [140,141]. Iron porphyrins can be linked to the highly porous network of MOFs, a promising avenue for strengthening overall ORR catalytic activity and stability. The open framework facilitates the flow of O_2_ to the active iron sites and the diffusion of H_2_O or H_2_O_2_. The iron porphyrin-based MOFs typically favor the 4e^−^ pathway, especially in acidic media [38,142]. The main advantage of introducing iron porphyrins into MOFs is the ability to maintain structural integrity. Unlike free iron porphyrins that can be degraded under reaction conditions, the iron porphyrin-based MOFs show improved durability due to the protective framework stabilizing the active sites [143,144,145].

**Figure 22:**
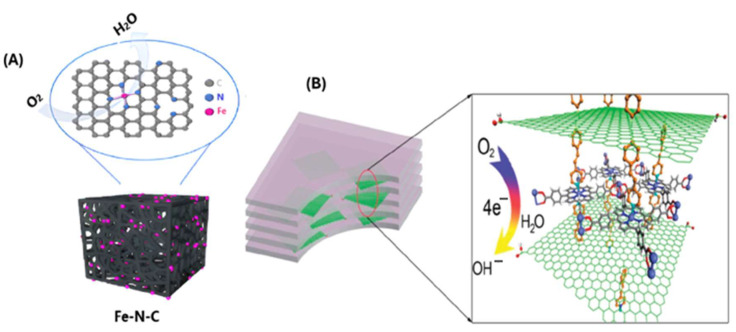
(**A**) The Fe-N-C catalyst. Reproduced from Ref. [146]. (**B**) Magnified view of layers inside the framework of graphene-porphyrin MOF composite. Reproduced from Ref. [147] with permission from Advanced Science, copyright 2012.

Quite a few studies highlight the benefits of attaching iron porphyrins to MOFs. Guo, X.-S. et al. developed an efficient iron–nitrogen–carbon (Fe-N-C) ORR catalyst via doping dicyandiamide (nitrogen source) and iron porphyrins (Fe^II^TPP) on a pyrolyzed MOF-5 square-shaped carbon material. Figure 22A shows the Fe-N-C catalyst. The Fe-N-C catalyst exhibited exceptional ORR performance, making it a promising alternative to platinum-based catalysts for fuel cells and zinc–air batteries. Under an alkaline environment, the Fe-N-C catalyst has E_onset_ and E_1/2_ values of 1.002 V and 0.903 V, respectively, out-performing platinum-based catalysts under the same conditions. The excellent activity is attributed to the iron active sites being uniformly dispersed on the squared structure carbon material. At the same time, the high specific surface area facilitates the exposure of the active sites. Additionally, the catalyst showed excellent stability and methanol resistance [146].

Jahan, M. et al. showed the efficacy of a graphene–porphyrin MOF composite for enhanced ORR electrocatalytic activity. Pyridine-functionalized graphene was synthesized and assembled with iron porphyrin to form the MOF composite. Figure 22B shows the magnified view of the graphene–porphyrin MOF composite. The study results show that the graphene–porphyrin MOF composite exhibits improved ORR performance with a facile 4e^−^ reduction process. The addition of the pyridine-functionalized graphene changes the crystallization process of iron porphyrin in the MOF, increases its porosity, and boosts the electrochemical charge transfer rate of iron porphyrin. Furthermore, the MOF composite showed good tolerance towards methanol and superior durability compared to other tested catalysts [147].

**Figure 23:**
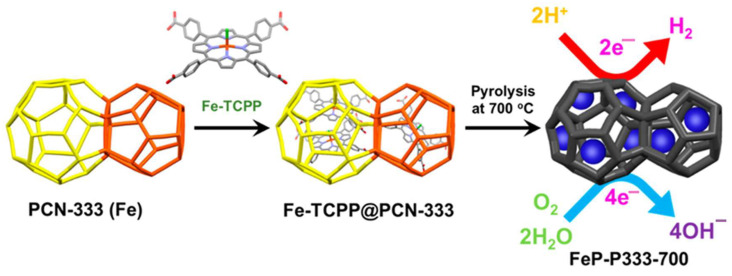
Preparation of FeP-P333-700 electrocatalyst. Reproduced from Ref. [148] with permission from Journal of the American Chemical Society, copyright 2017.

Another report, Park, J. et al. created an efficient electrocatalyst for the ORR and hydrogen evolution reaction (HER) using iron(III) porphyrin-encapsulated MOFs. The Fe/Fe_3_C-embedded nitrogen-doped carbon material (FeP-P333-700) was engineered via the pyrolysis of an iron porphyrin-encapsulated iron-based MOF (Fe-TCPP@PCN-333). Figure 23 illustrates the preparation of the catalyst. The Fe/Fe_3_C-embedded nitrogen-doped carbon material (FeP-P333-700) showcased superior electrocatalytic performance for ORRs and HERs resulting from the synergistic effect of Fe/Fe_3_C and Fe-N-C active sites. The superior ORR activity is comparable to commercial Pt/C [148].

The improvements in durability and catalytic efficiency described by reports on iron porphyrin-based MOFs suggest the feasible application of these materials as successors to the common catalyst. Incorporating iron porphyrin into MOFs leads to a synergistic system where the catalytic activity imparted by iron was appropriately integrated with the structural merits provided by the MOF scaffold. Compared with free iron porphyrin, which decomposes under harsh reaction conditions, iron active sites are stable for a long period of time in this robust MOF. The three-dimensional porous network also gives way to continuous accessibility to the active sites, allowing for the efficient diffusion of reactants and removal of products. It is very stable during the ORR; hence, it is very promising for use in practical energy conversion technologies.

However, there remain challenges regarding the structure’s scalability and maintenance of high conductivity throughout the MOF structure [140]. In the case of MOFs that incorporate complex iron porphyrins, their synthesis is a multistep process under stringent conditions; hence, scaling up is a complicated and costly task [149]. The search for less complex synthesis pathways that can easily be scaled up remains a key challenge for their commercialization. In this regard, further optimization of the incorporation method of iron porphyrins into the MOF for maximum stability and conductivity needs to be attempted. Approaches like modifying the linker molecules to enhance electrical conductivity or incorporating conductive elements in the MOF structure would greatly enhance catalytic performance. It could also be that research into novel types of MOFs with pre-designed pore sizes and specified metal centers might give even more perspective on catalyst efficiency and durability.

### 4.6. Heteroatom Doping Iron-Based Composites

Introducing heteroatoms such as nitrogen (N), boron (B), phosphorus (P), and sulfur (S) into the iron-based composites is a prominent trend in ORR research, focused on altering and improving the electronic environment around the iron center. This strategy influences the composite’s catalytic performance and durability for ORRs [150]. Doping can adjust the electronic structure of the electrocatalyst, meaning it can change the electron density surrounding the iron center, which may result in either an increase in electron density or a deficiency in electrons based on the specific dopant [151]. For example, doping with nitrogen typically establishes electron-rich sites around the iron center due to nitrogen’s significant electronegativity. The alteration can boost the electron density of iron, improving ORR performance by promoting electron transfer to oxygen molecules, improving their binding strength and lowering the activation energy for their reduction [152]. One study by Li, J. et al. shows the effectiveness of N-doping iron porphyrin. They developed a non-precious metal electrocatalyst (NPME) that consisted of N-doped graphene-like layers by carbonizing iron(III) porphyrin (FeP) that was supported on carbon black. The synthesized NPME displayed high ORR activity and durability in alkaline and acidic media, which was superior to Pt/C. It had a value of E_1/2_ of 0.87 V vs. RHE in alkaline medium and 0.75 V vs. RHE in an acidic medium. The NPME also showed a direct 4e^−^ ORR process in an alkaline medium [153].

Similarly, phosphorus and sulfur can modify the catalyst’s electron density and polarity due to their electron-donating nature, thereby increasing the reactivity of iron sites for ORRs by optimizing the adsorption and desorption processes of oxygen-related species. Furthermore, P-doping can improve catalyst stability, especially under acidic conditions, where phosphorous–carbon bonds can resist oxidative degradation. This stabilizing effect is valuable for long-term fuel cell applications [154,155].

In contrast, boron exhibits a lower electronegativity than carbon, forming electron-deficient areas within the composite. This variation can affect the spin state and electronic arrangement of the iron center, enhancing the adsorption of oxygen intermediates and, in turn, the ORR kinetics. Additionally, boron doping can enhance the durability of the composite by stabilizing the reactive sites [156,157,158].

Doping with heteroatoms enhances active sites by inducing defects or deformations in the composite structure, revealing additional active sites or generating new ones that are more accessible to reactants. This characteristic is vital for ORRs, as it boots the number of available catalytic sites and potentially improves overall activity [159,160]. When multiple dopants are used together, it frequently leads to synergistic effects that can further boost catalytic performance [152]. For instance, nitrogen may enhance conductivity, whereas sulfur can assist in fine-tuning the adsorption energy of intermediates, which together improves both catalytic efficiency and stability in acidic or alkaline conditions [161].

Incorporating these heteroatoms into the iron porphyrin composites will allow for precise adjustments to their electronic structure and catalytic efficiency, rendering them more competitive with platinum-based catalysts for ORRs. By thoughtfully choosing the type and concentration of the dopant, researchers can substantially improve ORR performance, enhancing activity, stability, and cost-effectiveness [162]. Potentially, these modifications to the composites can display better electron transfer and durability across various electrolytes, presenting promising opportunities for practical applications in fuel cells and metal–air batteries. This will drive the development of next-generation catalysts that are efficient, stable, and economically viable for sustainable energy solutions.

## 5. Platinum-Based Catalyst vs. Iron Porphyrin-Based Composites

Iron porphyrin-based composites have emerged as a promising substitute for platinum-based catalysts in ORR catalysis, driven by the demand for affordable, sustainable, and efficient catalysts for fuel cell uses. Although platinum is still regarded as the gold standard due to its excellent catalytic activity, its high cost, limited availability, and susceptibility to degradation in acidic conditions have led to significant research into alternative materials [28]. As highlighted, iron porphyrins have distinct advantages, such as adjustable structural properties that influence their electronic characteristics and the capacity to facilitate ORRs through the 4e^−^ route. Herein, this work evaluates specific major ORR performance indicators—including onset potential, half-wave potential, durability, and stability—of iron porphyrin-based composites compared to platinum.

i. Onset and Half-wave Potential. Platinum-based catalysts, particularly Pt/C, display onset potential, usually around 0.95 V vs. RHE in both acidic and alkaline electrolytes, with some advanced platinum alloys reaching slightly higher potentials. The high E_onset_ is linked to platinum’s strong binding with O_2_ and efficient activation of the O=O bond. The Pt/C half-wave potential is generally around 0.85–0.9 V vs. RHE, demonstrating faster ORR process kinetics [35,163,164]. These metrics highlight platinum’s effectiveness in lowering the activation energy needed for O_2_ reduction and promoting ORR efficiency. In comparison, modified iron porphyrin-based catalysts or those integrated into MOFs exhibit promising E_onset_ and E_1/2_ values, especially under alkaline conditions. For instance, complex **6,** when tested in 0.1 M KOH, shows an E_onset_ of 1.04 V and E_1/2_ of 0.87 V vs. RHE, comparable to Pt/C. Likewise, under an alkaline condition, the Fe-N-C catalyst shown in Figure 22A has E_onset_ and E_1/2_ values of 1.002 V and 0.903 V, respectively, out-performing platinum-based catalysts. However, the performance in acidic media still lags Pt/C due to degradation and stability issues.

ii. Electron transfer number. Iron porphyrins often favor the 4e^−^ pathway in alkaline and acidic media, which is desirable for efficient ORR. Likewise, Pt/c catalysts consistently achieve the 4e^−^ pathway, even across various pH values, yielding high selectivity for water production over peroxide intermediates [165].

iii. Durability and Stability. Pt-based catalysts are susceptible to long-term degradation, especially in acidic media [166]. Pt-Ni alloy catalysts demonstrate extended durability and stability due to the suppression of platinum oxidation. However, the Pt-Ni alloy still presents performance loss after extended cycling [167]. Similarly, the stability of iron porphyrin catalysts remains a primary concern, especially under acidic conditions, where rapid oxidative degradation occurs. They demonstrate better durability in alkaline conditions, often retaining 70–85% of their initial activity [168]. Strategies like incorporating porphyrin into MOFs or using nitrogen-doped carbon support have improved durability, although challenges remain for prolonged use under acidic conditions.

iv. Cost and Practicality. Despite its exceptional ORR activity, platinum’s high cost and limited availability prevent it from widespread adoption. Furthermore, platinum extraction and processing have significant environmental impacts, including greenhouse gas emissions and ecological disruptions [169]. For these reasons, the large-scale production of platinum catalysts for mass deployment would not be sustainable, especially for emerging green energy technologies. On the other hand, iron porphyrins are considered more economical, as iron is abundant and much less expensive than platinum. Additionally, using carbon-based supports, such as graphene, biochar and activated carbon, adds minimal cost [170,171].

Iron porphyrin-based composites are a viable and sustainable alternative to platinum-based catalysts for ORR applications. While platinum remains the benchmark for catalytic efficiency—generally demonstrating superior E_onset_ and E_1/2_ values and better durability in acidic and alkaline environments—its high cost, scarcity, and environmental impacts pose significant barriers to its large-scale application. In contrast, iron porphyrins offer an economically feasible solution, benefiting from the earth’s iron abundance and iron’s structural versatility, which allows for tailored electronic properties to promote ORRs. Although promising in alkaline media, iron porphyrin catalysts still face challenges in acidic conditions, where stability and durability concerns persist due to oxidative degradation. Recent advances, such as embedding iron porphyrin within MOFs and using heteroatom doping, have shown promise in improving their performance and longevity. Addressing these challenges and optimizing iron porphyrin, particularly for acidic applications, could bridge the performance gap with platinum, contributing to a new generation of cost-effective and environmentally sustainable fuel cell technologies.

## 6. Conclusions and Perspectives

The application of iron porphyrin-based components in the electrocatalytic mechanism for oxygen reduction reactions is a significant area of research due to their potential in energy conversion and storage technologies, particularly in fuel cells and metal–air batteries. This paper has established that iron porphyrin-based components are effective catalysts for ORRs due to their high catalytic efficiency, tunability, high redox flexibility, strong oxygen binding, and cost-effectiveness. From Table 1, a summary of catalytic performance by these complexes indicates that specific alterations and support materials are significantly related to their properties in affecting the nature of their electrochemical performance. However, despite the many excellent properties, iron porphyrin-based components face challenges like stability and degradation issues, scalability issues, and difficulty maintaining high conductivity, affecting their overall efficiency. For example, iron has durability and stability issues due to Fenton reactions occurring during the ORR process. Fenton reactions involve the generation of hydroxyl (OH^●^) and hydroperoxyl (OOH^●)^ radicals through the interaction of H_2_O_2_ with the Fe^2+^ ion, typically favored under acidic conditions, as shown below [18,172,173,174].

Fe^2+^ + H_2_O_2_ → Fe^3+^ + OH^●^ + OH^−^ (18)

Fe^3+^ + H_2_O_2_ → Fe^2+^ + OOH^●^ + H^+^ (19)

The hydroxyl and hydroperoxyl radicals can degrade the carbon support material for the catalysts and even dissolve the iron ion during the reduction reaction [85,173]. When utilizing iron materials for ORRs, finding ways to mitigate Fenton effects is crucial to improve stability. One possible solution is monitoring the pH levels of the electrolyte.

Another challenge facing iron porphyrin-based electrocatalysts is their susceptibility to leaching in acidic mediums. The leaching diminishes the number of active catalytic sites and shortens the electrocatalyst’s lifespan in practical applications. These stability concerns affect the durability and efficiency of the catalyst, and they will not be feasible for industrial applications without improvements.

To address these challenges, it is recommended that encapsulation techniques be developed to shield iron porphyrin catalysts from harsh operating conditions, improving stability and lowering the chances of degradation. Encapsulating the catalyst within MOFs or graphene structures is a promising approach. Iron porphyrins will also benefit from synthesis with more robust and stable ligands that can resist degradation, resist binding by reactive contaminants, and prevent iron leaching under operational conditions. This will enhance the oxidative and thermal stability of the catalysts. Also, there is a need to employ stable and conductive support materials such as activated carbon or carbon nanotubes to enhance the structural integrity and electron transfer capabilities of iron porphyrin catalysts, hence strengthening the stability, electrical conductivity, and catalytic performance of iron porphyrins. Notably, the focus should be on developing simplified and cost-effective synthesis methods that allow for the large-scale production of iron porphyrin catalysts without compromising quality and performance.

Implementing these recommendations to address the challenges of iron porphyrin-based components is essential for developing more efficient, durable, stable, and economically viable iron porphyrin catalysts for ORRs and advancing the application of iron porphyrin-based catalysts in energy conversion technologies.

## Figures and Tables

**Table 1:** Summary of the ORR catalytic performance of iron porphyrin-based composites.

Catalyst System	Electrolyte	Support Material	E_onset_ (mV)	E_1/2_ (mV)	Electron Transfer Number (*n*)	Ref.
Fe^II^TPP-CNT	0.1 M KOH	CNT	790 vs. RHE	640 vs. RHE	≈3.8	[44]
Complex **1**	0.1 M KPF_6_	EPG electrode	−200 vs. Ag/AgCl	-	-	[98]
Complex **2**	0.1 M KPF_6_	EPG Electrode	−300 vs. Ag/AgCl	-	-	[98]
Complex **3**	0.1 M KOH	MWCNTs	920 vs. RHE	770 vs. RHE	≈3.8	[47]
Complex **4**	0.1 M KOH	MWCNTs	910 vs. RHE	750 vs. RHE	≈3.6	[47]
Complex **5**	0.1 M KOH	MWCNTs	930 vs. RHE	810 vs. RHE	≈3.8	[47]
Complex **6**	0.1 M KOH	MWCNTs	1040 vs. RHE	870 vs. RHE	≈4.0	[47]
Complex **7**	0.1 M KOH	CNT	930 vs. RHE	840 vs. RHE	3.97	[57]
Complex **8**	0.1 M KOH	CNT	850 vs. RHE	680 vs. RHE	3.84	[57]
Complex **9**	0.1 M KPF_6_	Graphite disk	-	-	3.7 (pH 7)	[99]
Complex **10**	Phosphate buffer solution	EPG electrode	−240 vs. Ag/AgCl	−255 vs. Ag/AgCl	-	[100]
Complex **13**	0.5 M Triflic acid (HOTf)	-	400 vs. NHE	-	-	[101]
Complex **14**	0.5 M HOTf	-	500 vs. NHE	-	-	[101]
Complex **17**	0.1 M KPF_6_	EPG electrode	−250 vs. Ag/AgCl	-	-	[102]
Complex **18**	0.1 M KPF_6_	EPG electrode	−310 vs. Ag/AgCl	-	-	[102]
Complex **19**	0.1 M KPF_6_	EPG electrode	−300 vs. Ag/AgCl	-	-	[102]
Complex **27**	0.5 M H_2_SO_4_	-	880 vs. RHE	780 vs. RHE	≈4.0	[103]

## Data Availability

Not applicable.
